# An Autoencoder-Based Deep Learning Approach for Load Identification in Structural Dynamics

**DOI:** 10.3390/s21124207

**Published:** 2021-06-19

**Authors:** Luca Rosafalco, Andrea Manzoni, Stefano Mariani, Alberto Corigliano

**Affiliations:** 1 Dipartimento di Ingegneria Civile e Ambientale, Politecnico di Milano, Piazza L. da Vinci 32, 20133 Milano, Italy; stefano.mariani@polimi.it (S.M.); alberto.corigliano@polimi.it (A.C.); 2 MOX, Dipartimento di Matematica, Politecnico di Milano, Piazza L. da Vinci 32, 20133 Milano, Italy; andrea1.manzoni@polimi.it

**Keywords:** load/system identification, deep learning, structural dynamics, autoencoder, false nearest neighbor

## Abstract

In civil engineering, different machine learning algorithms have been adopted to process the huge amount of data continuously acquired through sensor networks and solve inverse problems. Challenging issues linked to structural health monitoring or load identification are currently related to big data, consisting of structural vibration recordings shaped as a multivariate time series. Any algorithm should therefore allow an effective dimensionality reduction, retaining the informative content of data and inferring correlations within and across the time series. Within this framework, we propose a time series AutoEncoder (AE) employing inception modules and residual learning for the encoding and the decoding parts, and an extremely reduced latent representation specifically tailored to tackle load identification tasks. We discuss the choice of the dimensionality of this latent representation, considering the sources of variability in the recordings and the inverse-forward nature of the AE. To help setting the aforementioned dimensionality, the false nearest neighbor heuristics is also exploited. The reported numerical results, related to shear buildings excited by dynamic loadings, highlight the signal reconstruction capacity of the proposed AE, and the capability to accomplish the load identification task.

## 1. Introduction

Modern sensor technology makes it possible to continuously monitor structures and infrastructures and, thank to the use of deep learning (DL) techniques, also an effective health management and structural prognostics [1]. The analysis of the vibrational response of buildings, i.e., of displacement and/or acceleration recordings shaped as time series (TS), is a popular approach to structural health monitoring (SHM) aiming to assess the damage state of the structures [2]. The said vibrational response depends both on the structural properties (mass and stiffness distribution, sources of damping, possible damage pattern), and on the environmental and operational conditions [3]. The identification of the loading conditions can improve the effectiveness of a SHM system allowing, e.g., the setting of physics-based models matching the structural frequencies to be monitored and not the harmonic components of the loading [4,5]. The importance of the topic has led, in the past, to a series of approaches addressing modal identification for output-only systems [6,7]. Due to the ill-posedness of the load identification problem, regularization methods based on singular value decomposition were proposed in [8]: they require a minimum tuning work to be applied, possibly within a multi-objective optimization framework [9]. Deterministic regularization methods have been recently adopted to quantify uncertainty as interval numbers [10], keeping the computational burden limited with respect to probabilistic approaches.

Similarly to what done by singular value decomposition-based methods, we exploit a reduced representation of vibrational data to deal with the ill-posedness of the load identification problem [11]. An AutoEncoder (AE) has been designed to perform the dimensionaliy reduction; such an AE represents the feature extraction task of the SHM approach [12]. An AE is a neural network (NN)-based architecture capable of performing nonlinear dimensionality reduction of its input [13]. Due to the difficulty in reconstructing the TS frequency content and peaks, AEs are normally employed after a pre-processing stage. In the SHM field, an AE was employed in [14] to reduce the dimensionality of measurements of vibrations induced by the vehicle-bridge interaction, preliminary transformed into the frequency domain, and exploited to quantify and localise damage. In [15], a sparse AE was used as regression model to identify the damage location in a seven-storey steel frame structure, performing dimensionality reduction through modal identification. Other paradigms were exploited for fault detection via signal reconstruction, e.g., through auto-associative extreme learning machines [16].

The advantages of employing DL to construct a regression model, after a preliminary data dimensionality reduction, were explored in [17,18]. In [19], contractive AEs were used to extract robust features and perform fault detection for a rotating machinery. Here, we also aim at exploiting the knowledge of the dynamic behaviour of the structure and its interaction with the loading conditions to highlight the connection between the reconstruction capacity of the AE and the properties of the structure under study.

Compared to classic regularization techniques, an AE and, more generally speaking, NNs require a training stage wherein the network is trained by means of a suitable dataset (training dataset). Such training of the AE allows us to handle large uncertainty levels, identifying hidden dependencies between the handled signals and the loading conditions. Dimensionality reduction is therefore intended to perform a data mining task [20]. Instead of applying a pre-processing stage before data dimensionality reduction, we directly operate on multivariate time series (MTS) to achieve at least two advantages: avoiding any feature engineering; obtaining a data reduced representation exploitable for different tasks.

A further aspect addressed in this paper is related to the optimal setting of the dimension of the latent representation; both the reconstruction capacity of the AE and the load identification task are indeed affected by the number of latent variables. As this number looks hard to establish a-priori, due to the highly nonlinear response of deep NNs, the false nearest neighbour (FNN) technique has been exploited. We show how this critical feature can be set a-posteriori, by looking at the activation of the latent variables.

Compared to the existing literature, the contributions of our work are manifold, and deal with: (i) the application of a DL framework for data dimensionality reduction and regression in view of load identification; (ii) the analysis of the impact of structural dynamics on the statistical content of the signals, thereby helping to understand the outcome of the AE training; (iii) the establishment of a criterion to set a suitably reduced number of latent variables.

The remainder of the paper is organized as follows. In Section 2, the AE architecture is described, with an eye on its use in the load identification task for structural dynamics. In Section 3, the a-posteriori choice of the dimensionality of the reduced data representation is addressed, showing how the FNN heuristics can be exploited (see also [21]). In Section 4, extensive numerical results are presented, focusing on shear buildings: first, a deep investigation is reported for a two-storey case; next, the 39-storey Pirelli Tower in Milan is considered to quantitatively assess the performance and the computational burden of the proposed methodology. Finally, Section 5 gathers some concluding remarks, and suggests future developments to ensure the applicability of the approach to other structural systems.

## 2. Autoencoders for Input (Load) Identification

### 2.1. Autoencoder Paradigm

In a NN architecture, weights and biases, collected in Ω, are set during the training stage by minimizing a loss function $c(\bar{U},U)$, where: $\bar{U}\in R^{L\times N^{out}}$ is the desired output, which is known within a supervised learning framework; $U=U(V,\Omega )\in R^{L\times N^{out}}$ is the NN output; V is the NN input. The definition of $c(U,\bar{U})$ depends on the task to be accomplished by the NN. In this work, as we aim at load identification, the loss function is given by the mean squared error (MSE):

(1) $$c(\bar{U},U)=\sum_{l=1}^{L}\sum_{n=1}^{N}{\left({\bar{u}}_{ln}-u_{ln}\right)}^{2},$$

where $u_{ln}$ is the (l,n)-th entry of U, and ${\bar{u}}_{ln}$ is the corresponding entry of $\bar{U}$. Loss minimization during training usually relies on the back-propagation of the error $c(\bar{U},U)$ (see, e.g., [22]); to achieve it, Adam [23], namely a first-order stochastic gradient descent algorithm, has been adopted.

Tailored to the mentioned load identification task, AEs are exploited to perform dimensionality reduction of the structural vibrations recorded by a sensor network. Within a time window (0,T), each sensor provides a univariate TS, while the entire collection of the recordings by the whole sensor network provides an MTS, that is a collection of synchronized TS. From now on, we denote by $V=[v_{1},\ldots ,v_{N}]\in R^{L\times N}$ the generic output of a monitoring system employing *N* sensors, each of them consisting of *L* samples within the considered time window.

Convolutional layers allow to detect both local correlations within a TS, and correlations among different TS. This latter aspect is very important for the SHM of civil infrastructures, as it provides a means to implicitly recognize the shape of the vibration modes [24,25]. By stacking convolutional layers, more complex correlation problems in time can be handled, allowing to detect the aforementioned modes of the structure, still keeping the number of the weights in Ω rather limited.

The designed AE stacks a first sequence of convolutional layers, which represent the encoder function enc:V→z, where $z\in R^{P}$ and P≪(L×N). The vector z, which is the latent representation of V, does not usually have a clear physical meaning, and requires a supervised procedure to interpret its content. This procedure, generally consisting of the recovery of the generative factors of the input dataset, can be accomplished only if few of the generative factors are known in advance [26]; in the following Section 3, the generative factors will be discussed in details.

**Figure 1:**
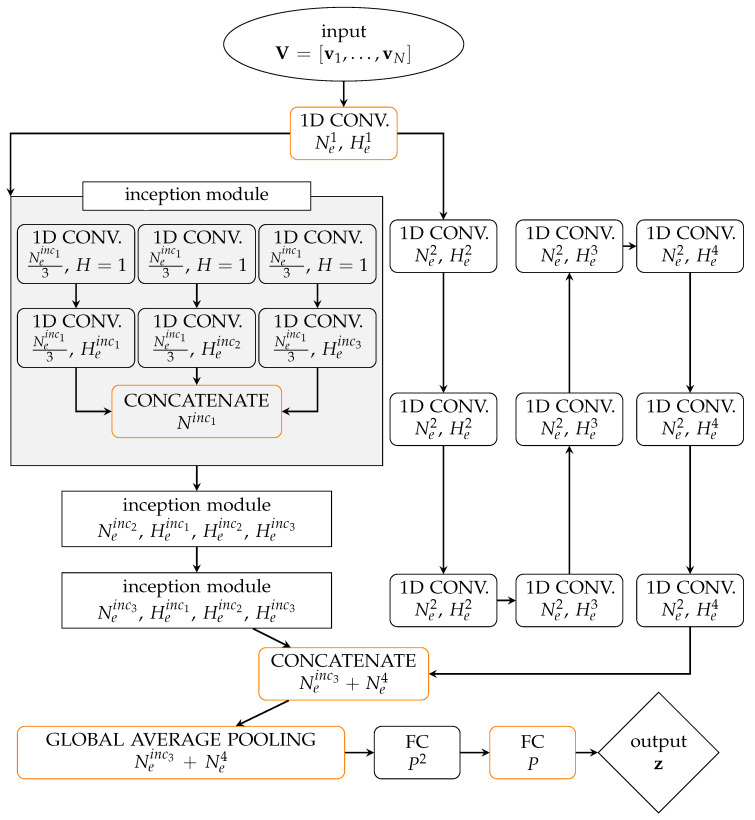
Architecture of the encoder. Layers are schematically represented by boxes: rectangular boxes with rounded corners depict single layer operations; rectangular boxes with sharp corners depict inception modules with dimensionality reduction; the elliptic box depicts the encoder input, while the diamond box depicts the encoder output. Orange edges are used for the layers that do not apply any activation function; for the other layers, the ReLU is used as activation function in the inception module, while the SELU is employed elsewhere. Each inception module assembles three one-dimensional convolutional (1D CONV.) layers and one concatenation layer, as shown in the grey box. In each box, the text in the first line specifies the layer type; the text in the second and (possibly) the third lines specifies instead the number of channels of the layer output and, for the convolutional layers, the kernel dimension.

The architecture of the adopted enc is depicted in Figure 1. It features two main branches: the first one stacking a sequence of three inception modules, operating dimensionality reduction [27]; the second one stacking nine one-dimensional convolutional layers, featuring a scaled exponential linear unit (SELU) activation function [28]. The two branches are preceded by a one-dimensional convolutional layer, and are followed by a concatenation layer, a global average pooling (GAP) layer [27] and, finally, two fully connected (FC) layers. Regarding these two final FC layers, the former employs a SELU activation function while the latter does not exploit any activation. All the convolutional layers employ a stride S=1. The inception modules have been proved beneficial to improve the reconstruction capacity of the AE, at variance with the residual learning paradigm (ResNet) [29] or the squeeze-and-excitation modules (SENet) [30]. The described architecture has been obtained after having explored several designs suited to pattern recognition.

We highlight that the reason behind the use of inception modules is the attempt of approximating the sparse deep NN structure for the optimal representation (intended as the capacity of inferring correlations) of the input dataset, see [31] for a theoretical discussion of this topic. Despite the lack of a rigorous mathematical proof connecting the peculiar design of the inception modules with the aforementioned theoretical evidences, inception modules have been proven successful in many applications. The same idea of approximating a sparse NN architecture by exploiting dense operators available in libraries like Tensorflow [32], here used with the Keras [33] API, has inspired the design of the employed two-branch architecture.

Through the GAP, synthetic information collected in a one-dimensional array is determined by computing an average value for each input channel. The last two FC layers are finally used to give extra flexibility to the encoder, to combine the synthetic descriptions of the channel contents extracted by the GAP and, as a result, to obtain z.

**Figure 2:**
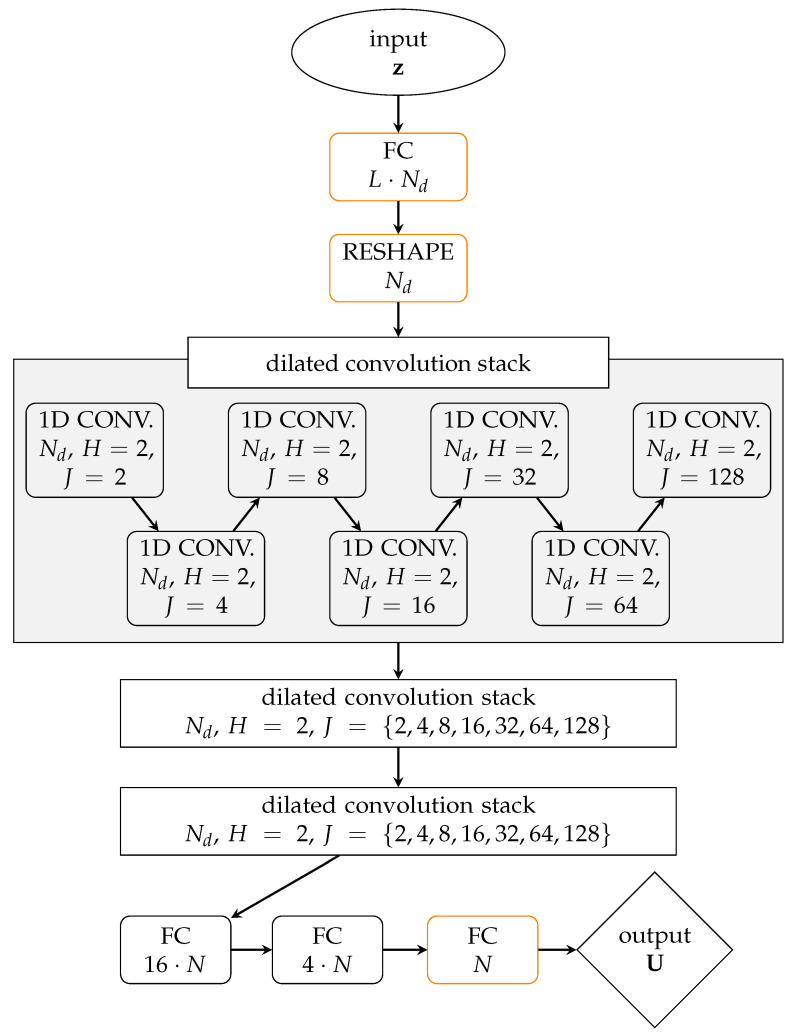
Architecture of the decoder. Layers are schematically represented by boxes, with the notation as detailed in the caption of Figure 1. Orange edges are used for the layers that do not apply any activation function, while the SELU is employed elsewhere. Each stack of dilated convolutions is formed by seven dilated convolutional layer, as shown in the grey box.

A second sequence of layers provides the subsequent decoder part, whose corresponding function is denoted by $dec:z\to \bar{U}$. Convolutional layers are also exploited for the decoder. The designed NN architecture of dec is depicted in Figure 2: first, a FC layer, employing no activation functions, is used to expand z; second, the one-dimensional array obtained with the FC layer, is reshaped as a two-dimensional array fashioned as an MTS; next, three convolutional sets are used, each of which stacking seven dilated convolutional layers; finally, three FC layers, the first two using a SELU activation function and the third without any activation, are employed to provide U. A dilated convolutional layer [34], with *H* setting the kernel of the convolution, operates by spacing its input with a dilation rate *J*. By setting J=1, a dilated convolution yields a standard convolution. The seven dilated convolutional layers feature H=2 and *J* doubled every layer up to J=128.

The use of three dilated convolution stacks have been inspired by [35]: there, it was argued that a stack of dilated convolutions, with *J* doubled every layer, is equivalent to apply a unique convolutional layer (with H=256), but in a by far more efficient way and involving much less parameters to tune. The intuition behind this architecture is that convolutions featuring smaller values of *J* are responsible for reconstructing short-term patterns, while those featuring larger *J* values allow the reconstruction of long-term patterns. Compared to [35], we have not employed causal connections, originally proposed to avoid the violation of the ordering of the layer input data, given the stationary framework of the present study. The last FC layers are used for combining the (possibly redundant) information contained in the output of the convolution stacks, reducing its dimensionality along the channel dimension.

**Figure 3:**
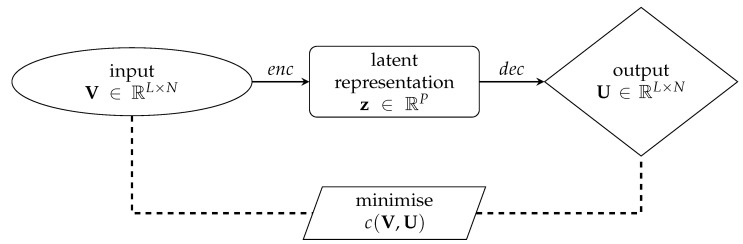
Schematic representation of the AutoEncoder. The loss function c(V,U) is computed as in Equation (1). For the enc and dec architectures, see Figure 1 and Figure 2, respectively.

If $\bar{U}$ has the same dimensions of V, the AE can accomplish the task of reconstructing the input V by setting $\bar{U}=V$ in Equation (1): the resulting working principle of the AE is sketched in Figure 3. The sought reduced representation z of the input V is thus obtained by composing the encoder and the decoder as dec∘enc:V→U, and setting U≈V.

### 2.2. Solving Regression Problems

The latent representation obtained with the proposed AE can be exploited for regression tasks [17], like load identification. Starting from the MTS V, assumed to be recorded by the deployed sensors, our goal is to determine the loadings applied to the structural system within the time window of interest. To this aim, we describe the loading conditions through a vector of parameters $\eta \in R^{Q}$; suitable probability density functions (pdfs) $P_{q}$ must be then associated to the entries $\eta_{q}$, q=1,…,Q. A similar approach was adopted in [36], where random variables, referred to as probabilistic input data, were used to describe the geometric properties and the parameters governing the carbonation process of a reinforced concrete beam.

**Figure 4:**
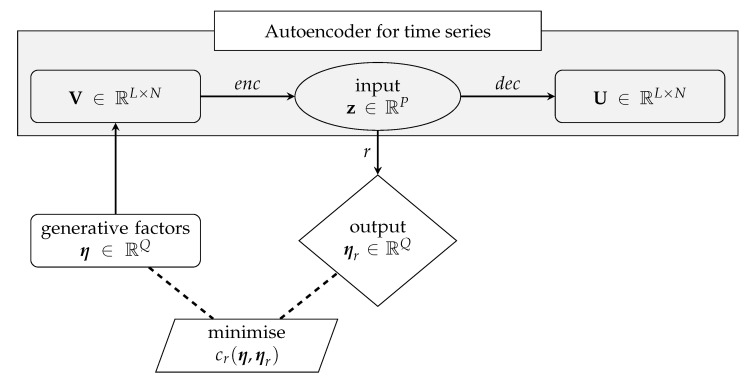
Schematic representation of the regression between z and η, wherein the loss function $c_{r}(\eta ,\eta_{r})$ is computed via Equation (2). For the enc and dec architectures, see again Figure 1 and Figure 2. For the *r* architecture, see Figure 5.

In the resulting problem, the independent regression variables are collected in V, while the dependent ones are collected in η. Using V as regression input allows to establish the dependence between η and the L×N variables in V or, in other words, to infer some relevant parameters ruling thousands of measurements of the structural response, without digging into the temporal correlation within each TS and the correlations among different TS. In this latter case, the use of a NN to accomplish the inference task, would require a large number of parameters in Ω to model η as η=η(V). For this reason, it would be way better to use z as regression input, being z a synthetic and exhaustive description of V, with *P* entries only. Figure 4 shows how the regression model r:z→η is integrated within the AE architecture.

**Figure 5:**
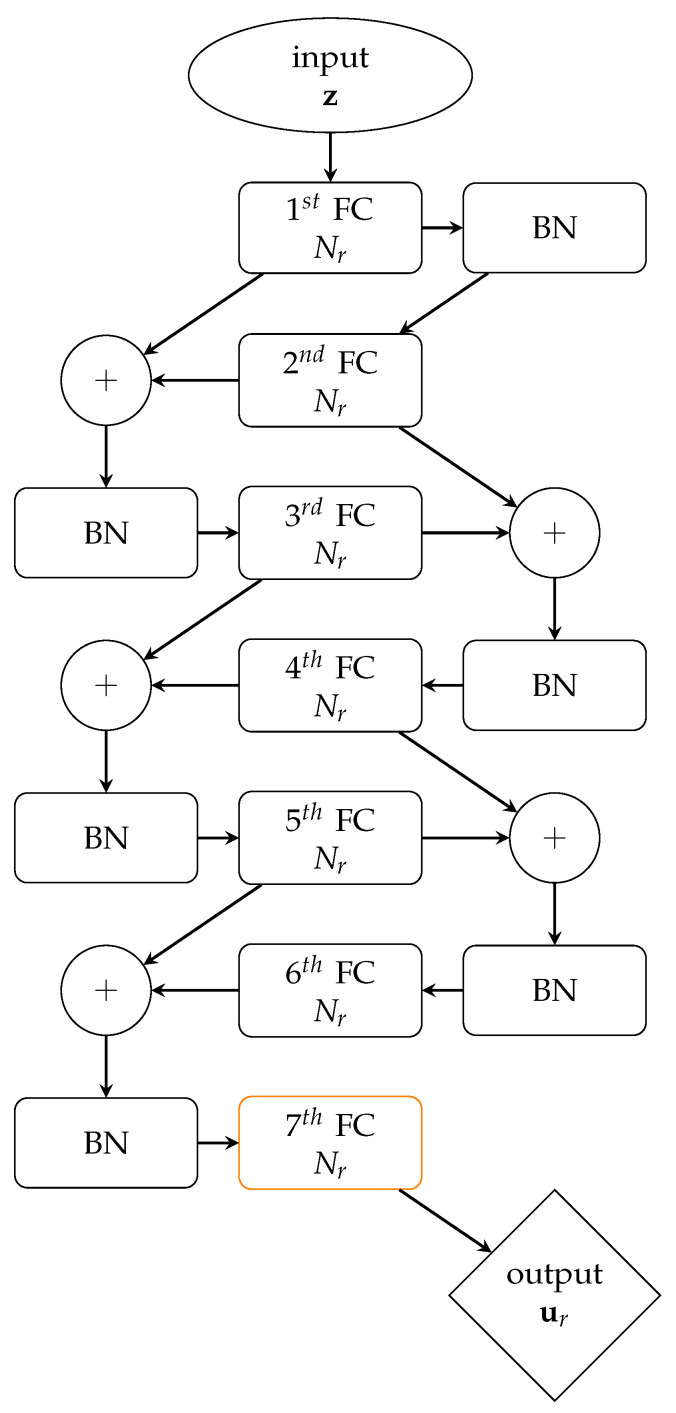
Architecture of the regression model; as for the notation, see the caption of Figure 1.

The designed NN architecture of the regression model *r* is depicted in Figure 5: first, six FC layers are stacked, each one featuring a SELU activation function and empowered by skip connections [29] and batch normalization (BN) [37]; then, a FC layer with no activation function provides the output $\eta_{r}$. Compared to the encoder and the decoder architectures, only one-dimensional arrays are involved in the layer definitions. Each FC layer outputs $N_{r}$ channels, and $N_{r}$ is therefore the only hyperparameter to tune in the proposed architecture.

Such a design has been inspired by the general trend to employ deeper and deeper NNs to minimize the loss $c_{r}(\eta ,\eta_{r})$, defined as:

(2) $$c_{r}(\eta ,\eta_{r})=\sum_{q=1}^{Q}{\left(\eta_{q}-\eta_{rq}\right)}^{2},$$

where η is the target output and $\eta_{r}\in R^{Q}$ is the output of the regression model.

Skip connections are employed to address the degradation problem usually suffered by deep NNs [38]. Denoting as $w:y_{i-1}\to y_{i}$ the transformation operated by the *i*-th FC layer, skip connections allow to obtain $\tilde{w}:y_{i-1}\to (y_{i}+y_{i-1})$, by summing $y_{i-1}$ (the *i*-th layer input) to $y_{i}$ (the *i*-th layer output). As discussed in [29], this usually enhances the training procedure, since it acts as a useful preconditioning of the mapping, so that the error can be more easily back-propagated through the whole NN. BN finally addresses the additional issue of the vanishing/exploding gradient problem, by zero-centering and normalizing the input.

We underline that the two training stages featured by this architecture are run separately: first, dec∘enc is trained by minimizing c(V,U); then, *r* is trained by minimizing the relevant cost function $c_{r}(\eta ,\eta_{r})$. A combined minimization of the two loss functions would make the training more complex, without enhancing the performance of the load identification task.

## 3. Choice of the Latent Dimension

### 3.1. Generative Factors

For the present study, if the mass and stiffness properties of the structural systems are assumed to be known, the structural frequencies and vibration modes are also set. The latent variables of the AE have to describe the time variability of the applied loads and, as a result, the structural response; the dimension *P* of z thus seems to be linked to η only, as this vector is responsible for the variability of the output and collects the generative factors. However, as it will be shown in the results Section, setting *P* according to this rationale does not prove to be a satisfactory option: to minimize the reconstruction error of the AE and to effectively solve the regression problem starting from the latent representation z, *P* must be increased. To understand how to set *P*, we first try to analyse separately how *enc* and *dec* work. Compared to other dimensionality reduction techniques, like principal component analysis, the AE implicitly requires to solve an inverse problem. More precisely, enc solves an inverse problem (find the latent factors z enabling the generation of the input V), while dec solves the specular forward problem, (reconstruct V from z). As seen previously, the inverse and forward problems are jointly solved by minimising the loss function c(V,U); it is thus necessary to train the NN in an unsupervised manner, without knowing a-priori z [17]. The solution of this problem may justify the need of adopting *P* greater than the least possible number of generative factors that one could assume just by considering the input dataset.

Indeed, as inverse problems are often ill-posed, a one-to-one correspondence between inputs in V and outputs in z usually does not exist.

### 3.2. False Nearest Neighbour Heuristics

From the discussion above, it seems hard to set a-priori the optimal number of latent variables, even when the number of generative factors for the dataset at hand is known. On one side, a too small value of *P* could have an impact on the decoder capability of reproducing V; on the other side, a too large value of *P* would introduce redundancy in the latent representation, that could ultimately spoil the minimization of c(V,U), making the understanding of the correlation between the latent variables and the model response even harder. It would be therefore useful to design a method to automatically set the optimal value of *P*.

Promising results in this regard come from the use of the FNN heuristics. Initially adopted for setting the appropriate embedding dimension of dynamic systems with the method of lags [39], the FNN heuristics has been recently proposed in [21] as activity regulariser for any NN employing hidden layers. The idea is to add a regularization term ϱ(z) to the AE loss function c(V,U), according to:

(3) $$c_{FNN}(V,U,z)=c(V,U)+\gamma L\varrho (z),$$

so that unnecessary activations of the latent variables are penalized. In Equation (3), γ>0 is an hyperparameter that sets the strength of the regularization term, while *L* is again the number of samples in each input TS.

If the variance of the activation values assumed by a latent variable becomes small, that variable will marginally affect the signal reconstruction, as if it were partially turned off. Therefore, once the AE is trained, the optimal number of latent variables is determined by looking at the latent variables featuring large activation variances for the dataset at hand. The crux is moved to the setting of a proper value for γ, an issue discussed in Section 4 and that we aim to further address in the future.

Coming to the regularization term, ϱ(z) is computed by counting the false nearest neighbors observed for different dimensions $P\leq P_{max}$ of the latent space, where $P_{max}$ is the maximum allowed dimension. The aforementioned counting is carried out by looking at the latent representation of each MTS V as a point in the space $R^{P}$, and evaluating if overlapping points in $R^{P}$ are well separated in $R^{P+1}$. If this is the case, these points are false neighbors in $\mathrm{span}\left\{z\right\}\in R^{P}$, and thus increase ϱ(z). A variational formulation of the FNN algorithm, originally proposed in [39], was developed in [21] to be compatible with the training of the NN via gradient descent-based techniques, and has been used in this work.

**Figure 6:**
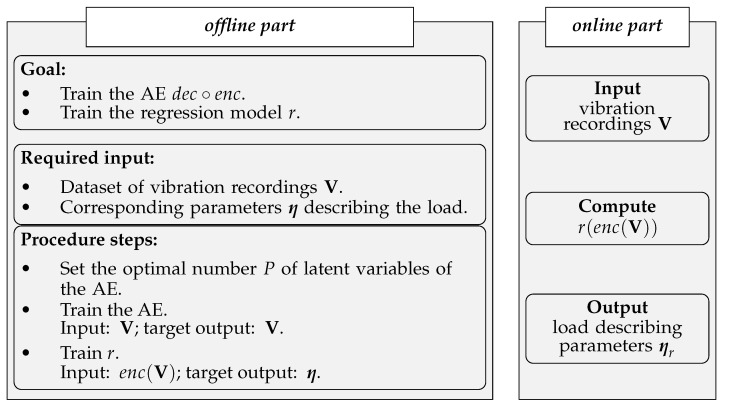
Proposed load identification strategy.

To clearly report the entire methodology here proposed, Figure 6 provides a recap of what discussed so far, addressing also the offline part of the procedure, namely the steps that must be taken before the use as a monitoring system.

## 4. Numerical Results

Two case studies are considered in the following, with the purpose of assessing the capability of the proposed approach to identify the parameters ruling the space and time variability of the loading applied to a structural system.

The first one deals with a two-storey shear building with lateral loads acting at each storey; it can be considered as the simplest possible structure of this type. Due to the limited need of computational resources to run the relevant structural model, a deep investigation is reported regarding the effect of the latent representation on the accuracy of the inverse problem solution, allowing also for the FNN heuristics.

The second one is the Pirelli tower in Milano, already studied, e.g., in [40,41,42], a cast-in-placed reinforced concrete building featuring 39 storeys for a total height of 130 m. Through this numerical case study, we show the feasibility of the proposed approach to deal with the difficulties arising from a real life high-rise building, still considering horizontal excitations only. The identification of the parameters ruling load amplitude and time variability has been performed in this case by exploiting the insights gained from the first case study.

### 4.1. Two-Storey Shear Building

#### 4.1.1. Shear Building Model

**Figure 7:**
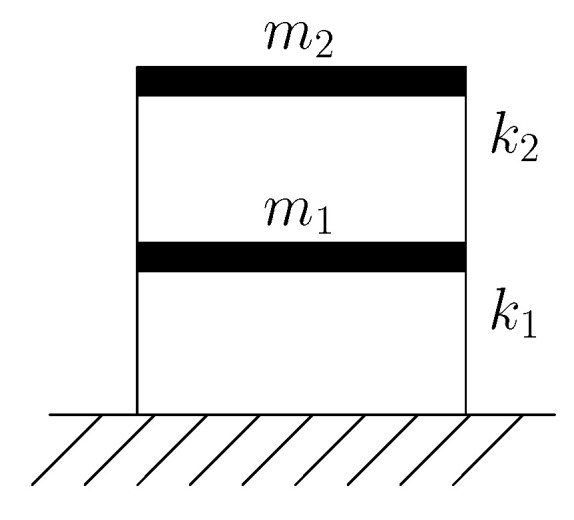
Two-storey shear building model. The horizontal, or lateral displacements are assumed to be recorded by the SHM system.

Shear building models are widely used in civil engineering for the vibration analysis of structures subject to lateral loads. Despite their simplicity, these models are very effective in characterizing the dynamic behavior of buildings whose floors have large out-of-plane stiffness. Their use in seismic analysis is encouraged by design codes like Eurocode 8 [43], that allow their adoption whenever requirements related to the aforementioned out-of-plane stiffness are satisfied, e.g., in terms of minimum slab thickness. To further simplify the analysis, we assume that the distribution of masses and stiffnesses are such that torsional effects can be neglected. Accordingly, the effects of lateral forces are decoupled along the two in-plan directions and the structural response of the building can be obtained by running two separate analysis, one for each lateral direction, by employing just one degree-of-freedom (dof) per floor. Damping effects have been disregarded, since they are usually not relevant in the identification of continuously excited systems [5,44]. A schematic representation of the considered two-storeys shear building model is reported in Figure 7.

The time dependent lateral loads $F(t)\in R^{L\times N}$ are applied to the floors, with a linearly increasing amplitude along the height of the building and a sinusoidal variation in time, namely

(4) $$\begin{array}{c} F_{n}(t)=\frac{n}{N}\alpha \sin(2\pi \phi t)\;\;n=1,\ldots ,N, \end{array}$$

where *t* is time; *N* is the number of floors (here N=2); α is the load amplitude factor, and ϕ is the load frequency. The resulting parameter vector is thus η={α,ϕ}, with Q=2 to fully describe each loading condition. To define the load, we have taken inspiration from the lateral force method described in [43], which can be adopted whenever the structural response is mainly related to the first mode of vibration of the building. The lateral force method can be adopted to define the peak actions in structural members through an equivalent static analysis under earthquake excitation; in our dynamic analyses, we have used the same variation of the load amplitude along the vertical direction. The sinusoidal variation in time has been instead adopted by considering that any dynamic loading can be decomposed into the sum of sinusoidal components through a Fourier series.

By assuming that the loading parameters α and ϕ, the floor masses and the inter-storey stiffnesses are constant in time, the building response is stationary within the time window of interest. If within each MTS the building always features the same mass and stiffness properties, long-term degradation effects like concrete carbonation and rebars rusting, chloride and sulphate attacks for reinforced concrete are disregarded. A discussion on how to cope with the effects of the mentioned degradation processes within a SHM procedure like the one here proposed, is beyond the scope of this work. Anyhow, it is worth stressing that our procedure will be, in principle, able to address this issue by including in the generative factors the stiffness reduction of the structural members, hence by modelling the effect of damage on the building response [24,25,45].

To identify the loads applied on a two-storey shear building, the dynamic response of the structure is numerically simulated and pseudo-experimental sensor recordings, shaped as MTS to form the input dataset, are represented by the storey lateral displacements.

#### 4.1.2. Signal Reconstruction

The AE has been first employed to reduce the dimensionality of the pseudo-experimental vibrational recordings. Each MTS $V\in R^{250\times 2}$ consists of two TS reporting the sampled time evolution of the lateral floor displacements for T=5 s, with a sampling rate of Δt=0.02 s. Each V is associated to a loading condition, whose governing parameters α and ϕ are sampled from two uniform pdfs $U_{\alpha }(0.625\times 10^{3},6.25\times 10^{3})\;N$ and $U_{\phi }(1,15)\;\mathrm{Hz}$. Two different building configurations, termed A and B and characterized by different vibration frequencies, have been considered to assess the effects of the interaction between the mentioned structural vibration frequencies and the accuracy of the results of the AE-based identification procedure. In Table 1, the data related to the two configurations are collected; note that the chosen sampling frequency for the pseudo-experimental measurements avoids signal aliasing to occur.

To train the AE, 16,000 MTS have been generated for both the configurations: 75% of these samples have been used to train the AE and back-propagate the error; the remaining 25% have been employed as validation set. Based on the loss c(V,U) computed on the validation set, an early-stopping strategy may be exploited. The training of the AE has been started from scratch for both configurations A and B, hence without relying on transfer learning.

For testing the trained AE, a further test set of 512 MTS has been generated. The reconstruction capacity of the AE is assessed not only qualitatively but also quantitatively, by computing for each MTS the two error measures reported in Table 2, and the relevant mean values and scattering around it over the whole training, validation and test sets.

Results in the following refer to AE hyperparameters gathered in Table 3: training has been repeated for different sets of hyperparameters, and the one adopted led to the minimum loss c(V,U) and has been finally selected for the analyses. As for the kernels of the convolutional layers, their dimensions have been set to cope with the fundamental period of structural vibrations, so as to exploit the capacity of convolutional layers to detect a correlation within a TS. Alternatives to this trial-and-error procedure are represented by the Bayesian methods [46] or by multi-objective optimization [47], which are anyway computationally infeasible in practical situations of interest for our study. The drawbacks of both approaches may be mitigated by their combination, as suggested in [48].

**Figure 8:**
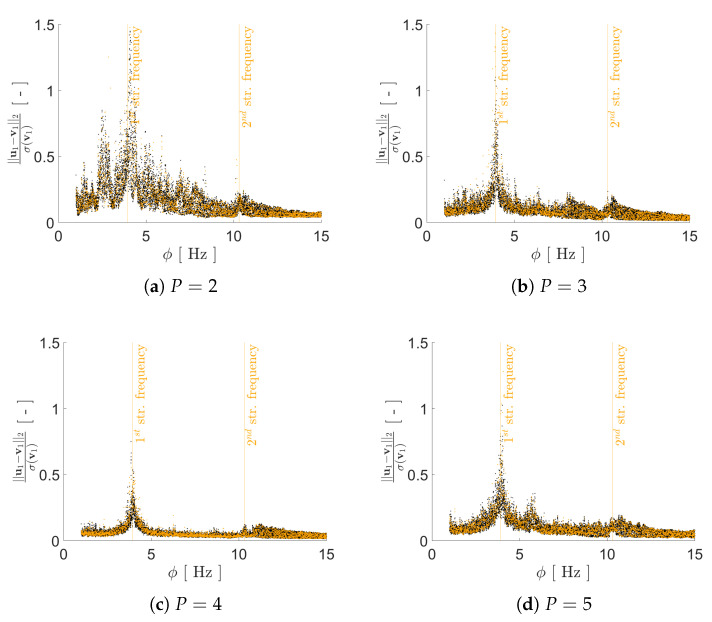

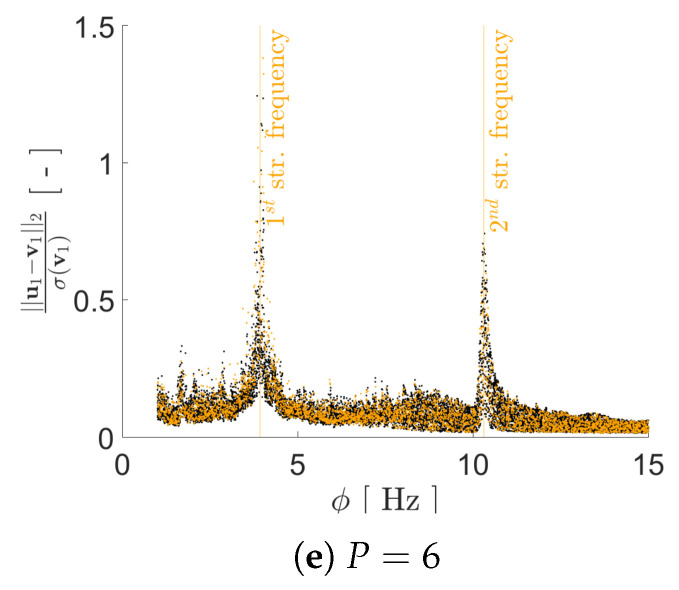
Two-storey shear building, configuration A. Reconstruction error for floor displacement $v_{1}$ via the standardized $L^{2}$ norm, as a function of the load frequency ϕ and for a varying value of *P*. In the charts, black dots refer to the training set, while orange dots refer to the validation set.

**Figure 9:**
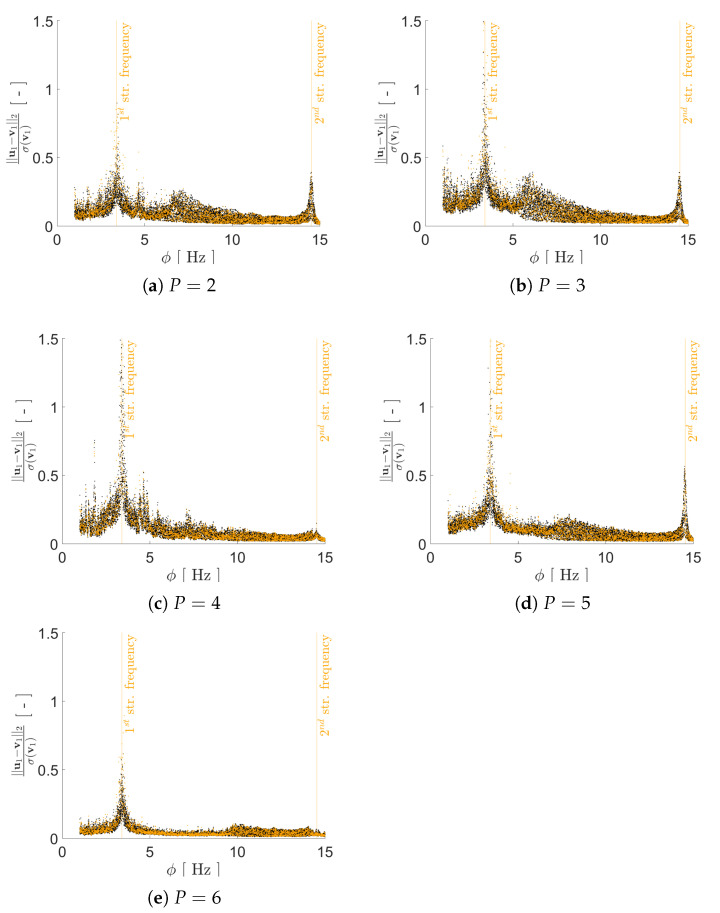
Two-storey shear building, configuration B. Reconstruction error for floor displacement $v_{1}$ via the standardized $L^{2}$ norm, as a function of the load frequency ϕ and for a varying value of *P*. In the charts, black dots refer to the training set, while orange dots refer to the validation set.

**Figure 10:**
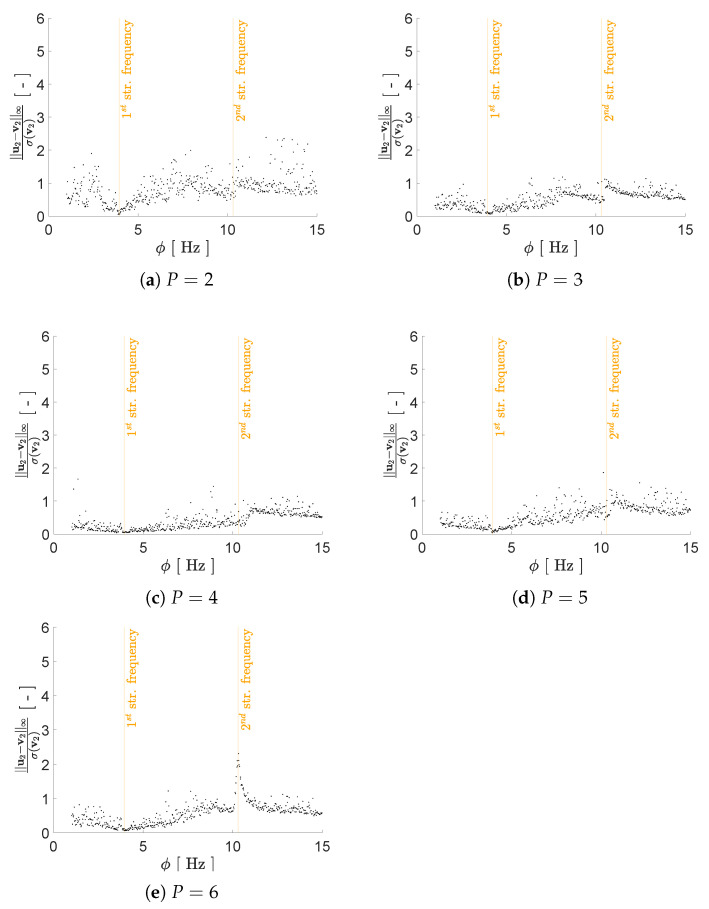
Two-storey shear building, configuration A. Reconstruction error for floor displacement $v_{2}$ via the standardized $L^{\infty }$ norm, as a function of the load frequency ϕ and for a varying value of *P*. In the charts, the dots refer to the test set.

**Figure 11:**
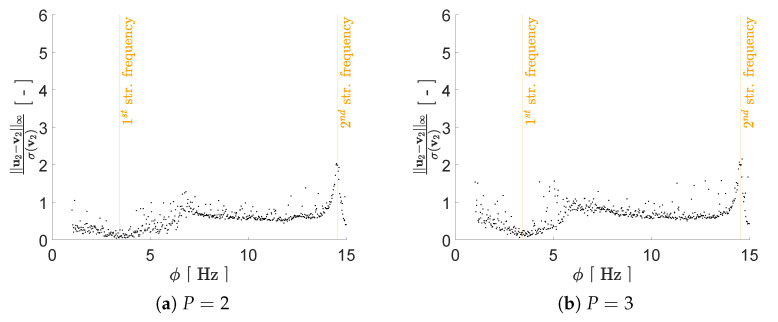

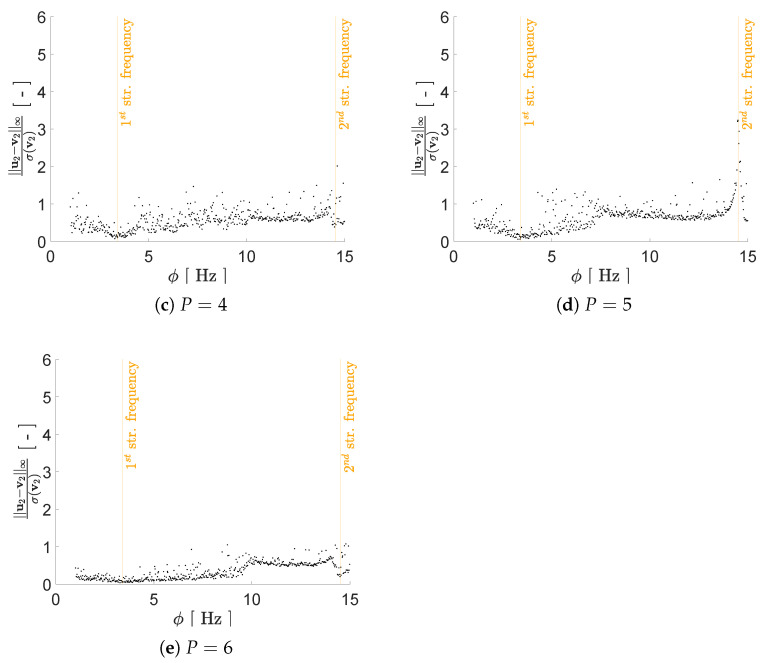
Two-storey shear building, configuration B. Reconstruction error for floor displacement $v_{2}$ via the standardized $L^{\infty }$ norm, as a function of the load frequency ϕ and for a varying value of *P*. In the charts, the dots refer to the test set.

**Figure 12:**
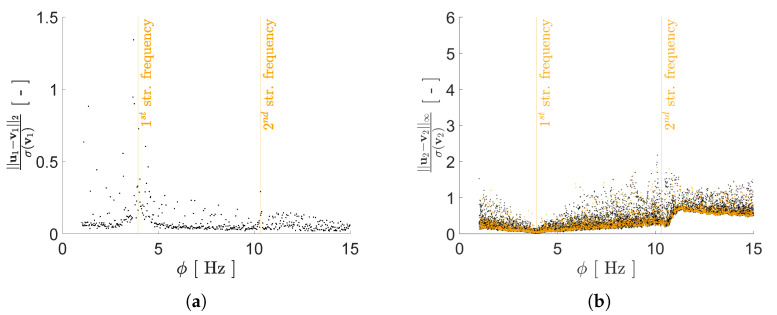
Two-storey shear building, configuration A, P=4. Reconstruction errors as a function of the load frequency ϕ: (**a**) standardized $L^{2}$ norm relevant to the floor displacement $v_{1}$ for the test set; (**b**) standardized $L^{\infty }$ norm relevant to the floor displacement $v_{2}$ for the training (black dots) and validation (orange dots) sets.

**Figure 13:**
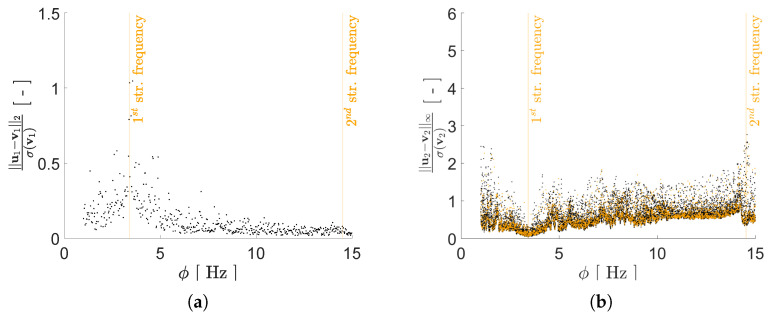
Two-storey shear building, configuration B, P=4. Reconstruction errors as a function of the load frequency ϕ: (**a**) standardized $L^{2}$ norm relevant to the floor displacement $v_{1}$ for the test set; (**b**) standardized $L^{\infty }$ norm relevant to the floor displacement $v_{2}$ for the training (black dots) and validation (orange dots) sets.

Regarding the impact of *P* on the reconstruction capacity of the AE, it has been assessed by means of its effects on mimicking the structural response in the frequency domain, starting from the reduced representation. As previously discussed, a lower bound on *P* can be assumed to be equal to 2, i.e., equal to the number of generative factors in η. Given the ill-posedness of the inverse problem, by adopting P>2 we expect to have beneficial effects on the AE performance; therefore, we have tested the cases P={2,3,4,5,6}. As shown in Figure 8, Figure 9, Figure 10 and Figure 11 the reconstruction error gets progressively reduced by increasing *P* for both the considered configurations, but unevenly and showing different correlations with the load frequency ϕ. This behavior is due to the stochastic nature of the training algorithm and also to the strong nonlinearity of c(V,U). More specifically: Figure 8 and Figure 9 report the error, via the standardized $L^{2}$ norm, in reconstructing the displacement $v_{1}$ of the first floor as a function of ϕ, when the input signals, respectively, belong to the training and validation sets; Figure 10 and Figure 11 report instead the error, via the standardized $L^{\infty }$ norm, in reconstructing the displacement $v_{2}$ of the second floor, when the input signals belong to the test set only. For comparison with these plots, Figure 12 and Figure 13 further provide, for configurations A and B, a sketch of the reconstruction capacity for the training and validation sets via the standardized $L^{\infty }$ norm, and a sketch of the reconstruction capacity for the test set via the standardized $L^{2}$ norm, both for P=4. Similar results have been obtained for the other values of *P*, but are not reported here for the sake of brevity. Such results are shown since, if the reconstruction capacity for the training and validation sets were greater than the one related to the test set, overfitting would have probably spoiled the AE performance: the NN would not acquire any generalization capacity, being limited to reproduce the instances seen during the training.

**Figure 14:**
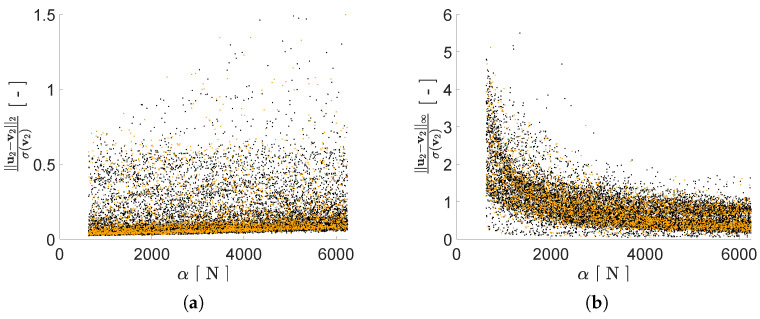
Two-storey shear building, configuration A, P=4. Reconstruction errors as a function of the load amplitude α, relevant to the floor displacement $v_{2}$ for the training (black dots) and validation (orange dots) sets: (**a**) standardized $L^{2}$ norm; (**b**) standardized $L^{\infty }$ norm.

The investigated reconstruction capacity is less affected by the load amplitude α, as shown in Figure 14, due to the linearity of the structural behavior. Indeed, when the standardized $L^{\infty }$ norm is considered, as it measures the inaccuracy in the peak reconstruction, larger errors are found for values of α smaller than 2000 N. In spite of the data normalization procedure preceding training, the structural displacements under excitations featuring small values of α have small peaks too, and their incorrect reconstruction results less penalized during the training.

The link between the reconstruction error and the load frequency ϕ varies with *P*, and depends on the adopted error measure. Figure 8 and Figure 9 have shown that the standardized $L^{2}$ error is larger when ϕ gets closer to the structural vibration frequencies $f_{1}$ and $f_{2}$, that is when the load induces a resonant response of the structure. This outcome is somehow expected, as the relevant beats in the displacement recordings are signal characteristics hard to catch by the AE. The larger error found for $\phi \approx f_{1}$ results as a consequence of the dec difficulty of reproducing the long-range temporal correlation characterizing the first vibration mode.

Figure 10 and Figure 11 have shown instead that the standardized $L^{\infty }$ error is still large when ϕ gets close to the second structural vibration frequency, while becomes rather small, roughly by ten times, for $\phi \approx f_{1}$. An analysis of the dynamics of the two configurations suggests the reason behind this result. During training, the loss function allows modifying more largely the weights Ω, when the AE fails to reconstruct the vibration mode that has a larger impact on the dynamic response of the structure. The excitation frequency ϕ is sampled from $U_{\phi }$ and the mentioned modes can have a different impact for different instances. To compute the impact that the vibration modes $\psi_{s}$, s=1,2, have on the solution, we first solve a non-standard eigenvalue problem in the form [49]:

(5) $$[K-{\left(2\pi f_{s}\right)}^{2}M]\psi_{s}=0$$

the stiffness and mass matrices of the structure being:

$$K=[\begin{array}{cc} k_{1}+k_{2} & -k_{2}\\ -k_{2} & k_{2} \end{array}]\;\;M=[\begin{array}{cc} m_{1} & 0\\ 0 & m_{2} \end{array}],$$

and enforce $\psi_{s}^{T}M\psi_{s}=1$ as normalization rule.

The equations governing the dynamics of the structure read:

(6) $$M\ddot{v}(t)+Kv(t)=F(t),$$

where: $\ddot{v}(t)\in R^{2}$ and $v(t)\in R^{2}$ are the vectors of storey accelerations and displacements, respectively; F(t) is the vector of the external loads. For each load case, by sampling v(t) at the two floors, we obtain $v_{1}$, $v_{2}\in R^{250}$ and the instance $V=[v_{1},v_{2}]$.

Due to the linear behavior of the structure, through modal superposition Equation (6) is decoupled as follows:

(7) $$\psi_{s}^{T}M\psi_{s}{\ddot{x}}_{s}(t)+\psi_{s}^{T}K\psi_{s}x_{s}(t)=\psi_{s}^{T}F(t),$$

with:

(8) $$v(t)=\psi_{1}x_{1}(t)+\psi_{2}x_{2}(t).$$

Since $\psi_{s}^{T}M\psi_{s}=1$, $\psi_{s}^{T}K\psi_{s}={\left(2\pi f_{s}\right)}^{2}$ and we obtain:

(9) $${\ddot{x}}_{s}(t)+{\left(2\pi f_{s}\right)}^{2}x_{s}(t)=\psi_{s}^{T}F(t).$$

If the structure is initially at rest and if the entries of the load vector F are defined according to Equation (4), the time history of $x_{s}(t)$ is given by:

(10) $$x_{s}(t)=\frac{\alpha }{{\left(2\pi f_{s}\right)}^{2}-{\left(2\pi \phi \right)}^{2}}(-\frac{\phi }{f_{s}}\sin(2\pi f_{s}t)+\sin(2\pi \phi t))\Gamma_{s},$$

where:

(11) $$\Gamma_{s}=\psi_{s}^{T}[\begin{array}{c} 0.5\\ 1 \end{array}]$$

actually depends on the structural dynamics (through $\psi_{s}$) and on the spatial distribution of loads.

At a specific time instant $\bar{t}$, the modal response becomes $x_{s}(\bar{t})={\bar{x}}_{s}$, whose expected value $E[{\bar{X}}_{s}]$ can be computed as:

(12) $$E[{\bar{X}}_{s}]=\int_{\alpha_{m}}^{\alpha_{M}}\int_{\phi_{m}}^{\phi_{M}}\{\frac{\alpha }{{\left(2\pi f_{s}\right)}^{2}-{\left(2\pi \phi \right)}^{2}}(-\frac{\phi }{f_{s}}\sin(2\pi f_{s}\bar{t})+\sin(2\pi \phi \bar{t}))\Gamma_{s}\;U_{\alpha }U_{\phi }\}d\alpha \;d\phi .$$

where we have accounted for that α and ϕ, respectively, vary in the ranges $\left(\alpha_{m},\alpha_{M}\right)$ and $\left(\phi_{m},\phi_{M}\right)$. Computing the integrals, we obtain:

(13) $$\begin{array}{cc} E[{\bar{X}}_{s}] & =\{\frac{\alpha_{M}-\alpha_{m}}{2}(\frac{\sin(2\pi f_{s}\bar{t})}{8\pi^{2}f_{s}}\ln\frac{f_{s}^{2}-\phi_{M}^{2}}{f_{s}^{2}-\phi_{m}^{2}}+I)\}\Gamma_{s}, \end{array}$$

where:

$$I=\int_{\phi_{m}}^{\phi_{M}}\frac{\sin(2\pi \phi \bar{t})}{4\pi^{2}(f_{s}^{2}-\phi^{2})}\;d\phi .$$

The term within curly brackets in Equation (13) provides the dependence of $E[{\bar{X}}_{s}]$ on α and ϕ.

At the same time instant, the expected value of the storey displacement ${\bar{v}}_{n}=v_{n}(\bar{t})$, n=1,2, thought of as sampled from the corresponding pdf ${\bar{V}}_{n}$, is obtained by exploiting Equation (8) and the linearity of the expectation rule [50]:

(14) $$E[{\bar{V}}_{n}]=\psi_{1n}E[{\bar{X}}_{1}]+\psi_{2n}E[{\bar{X}}_{2}].$$

The contribution to $E[{\bar{V}}_{n}]$ of each mode depends linearly on $E[{\bar{X}}_{1}]$ and $E[{\bar{X}}_{2}]$, and therefore on $\Gamma_{1}$ and $\Gamma_{2}$. For the case at hand, the ratio between $\Gamma_{1}$ and $\Gamma_{2}$ is equal to 9.67 for configuration A, and to 7.33 for configuration B. Accordingly, the error provided by the AE in reconstructing the contribution of the first vibration mode is, on average, roughly ten times larger than the error linked to the second vibration mode. The loss function c(U,V) leads to the setting of the NN weights in the same way. Due to this rationale, the AE is driven to learn better the first vibration mode.

In such a discussion, we have disregarded the temporal dependence of v(t); this has an impact on the AE capacity of accounting for each mode of vibration. In the comment to Figure 8 and Figure 9, we have already addressed that the tendency to learn better the first mode of vibration is counterbalanced by the long range temporal correlation featured by the first mode. The adopted error measures have been introduced with the purpose of investigating these issues, and seem to adequately address them.

**Figure 15:**
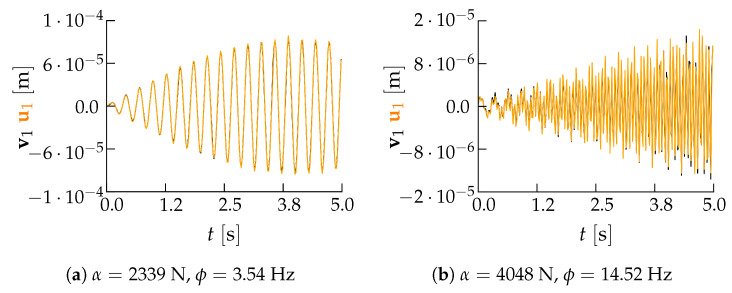
Two-storey shear building, configuration B, P=6. Comparison between input (black) and reconstructed (orange) time histories of the first floor displacement for two cases belonging to the validation set and for load frequencies (**a**) $\phi \approx f_{1}$ and (**b**) $\phi \approx f_{2}$.

Figure 15 shows a comparison of the reconstructed $u_{1}$ and the input $v_{1}$ signals taken from the validation set, either for $\phi \approx f_{1}$ or $\phi \approx f_{2}$ of configuration B and for P=6, to further get insights into what the two error norms provide. In spite of the rather large reconstruction error measured by the standardized $L^{2}$ norm and shown by Figure 9e for the first resonant frequency, $u_{1}$ and of $v_{1}$ in Figure 15a are almost perfectly superposed. This comparison confirms that both error measures bring meaningful information, with the standardized $L^{2}$ norm measuring inaccuracies in the reproduction of the frequency content of the input signal, while the standardized $L^{\infty }$ norm highlighting the inability to catch peaks in the same input signal.

**Figure 16:**
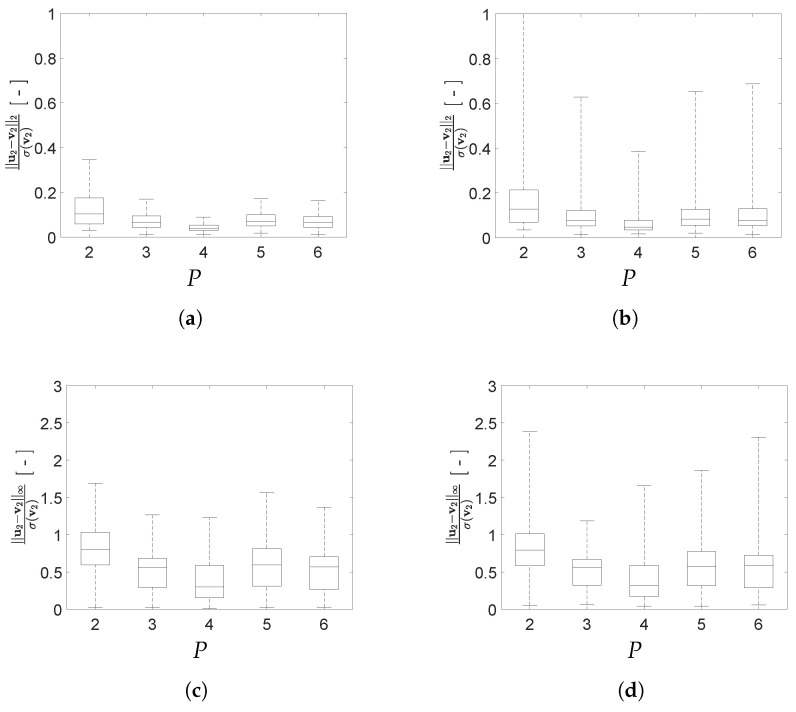
Two-storey shear building, configuration A. Effect of *P* on the statistics of the reconstruction errors relevant to the floor displacement $v_{2}$, represented through box plots: (**top row**) standardized $L^{2}$ norm, and (**bottom row**) standardized $L^{\infty }$ norm; data taken from (**left column**) training set, and (**right column**) test set.

**Figure 17:**
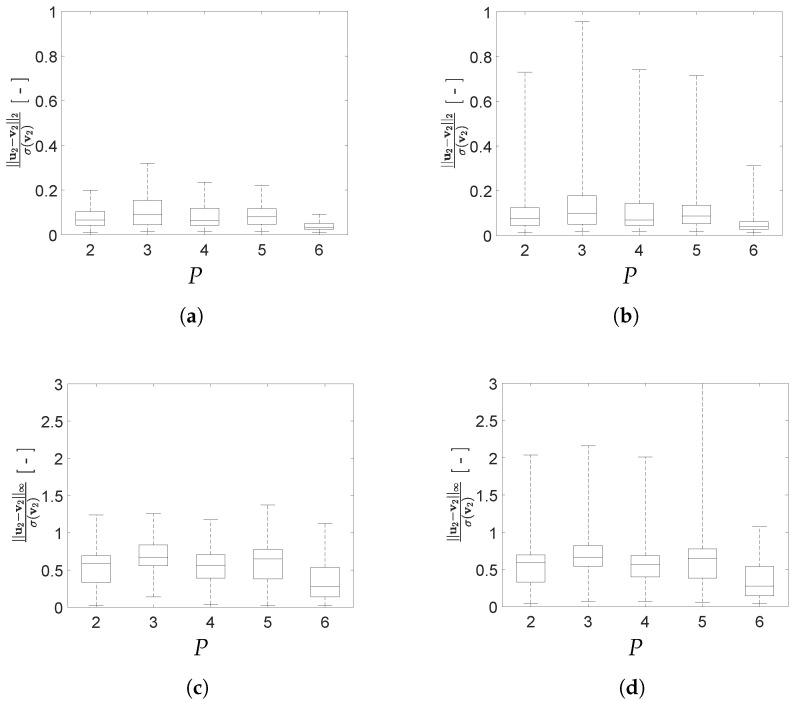
Two-storey shear building, configuration B. Effect of *P* on the statistics of the reconstruction errors relevant to the floor displacement $v_{2}$, represented through box plots: (**top row**) standardized $L^{2}$ norm, and (**bottom row**) standardized $L^{\infty }$ norm; data taken from (**left column**) training set, and (**right column**) test set.

To better assess the impact of *P* on the reconstruction capacity of the AE, box plots depicting the mean and the scattering around it for the two adopted error norms are reported in Figure 16 and Figure 17 for configurations A and B, respectively. In the charts, errors are given for both the training and test sets, to evaluate the generalization capacity of the AE. As a general rule, the values of the load configuration-dependent reconstruction error evaluated for the test set is more scattered than the one evaluated for the training set, while the relevant median values are quite similar; the said difference is larger if measured through the standardized $L^{2}$ norm.

According to Figure 16, the optimal number of latent variables for configuration A results to be P=4 when looking at the standardized $L^{2}$ norm, and P=3 when looking at the standardized $L^{\infty }$ norm if outliers are also allowed for. By increasing *P* and, therefore, the redundancy in the latent representation, an improvement of the AE reconstruction capacity is not achieved. As shown in Figure 17, also for configuration B an increase of the value *P* does not lead to a monotonic reduction of the reconstruction error. Even if the best AE accuracy has been obtained for P=6, good performances have been attained with P=4 too, with a slight deterioration for P=5.

**Figure 18:**
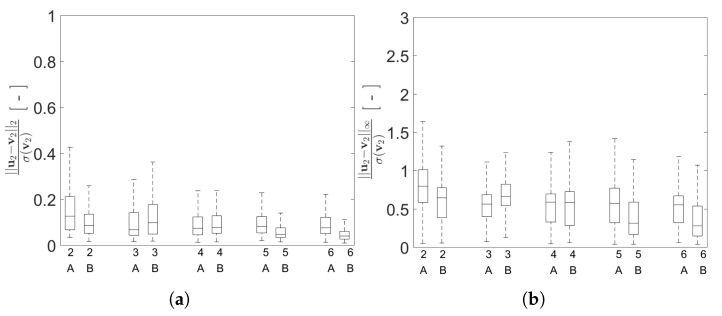
Two-storey shear building, effect of *P* on the statistics of the reconstruction errors relevant to the floor displacement $v_{2}$, represented through box plots, for data taken from the test set and both structural configurations A and B: (**a**) standardized $L^{2}$ norm; (**b**) standardized $L^{\infty }$ norm.

Moving deeper into the assessment of the AE performances, a comparison is reported in Figure 18 between the reconstruction errors for both configurations A and B. For the standardized $L^{2}$ norm, the variation relevant to the error values for configuration B is slightly smaller than that relevant to configuration A. A similar trend can be recognized also for the standardized $L^{\infty }$ norm. This outcome can be linked to the smaller gap between the resonance frequencies $f_{1}$ and $f_{2}$ featured by configuration A; it is worth mentioning that a similar difficulty was already observed with methods like indipendent component analysis or second order blind identification, when the identification of closely spaced modes is involved [51].

#### 4.1.3. False Nearest Neighbour Heuristics

**Figure 19:**
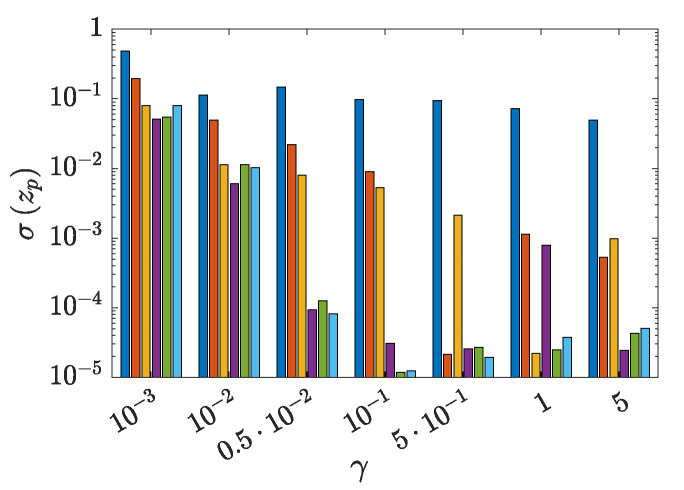
Two-storey shear building, configuration A. Effect of the regularization parameter γ on the value of the variance of each latent variable in z, evaluated on both the training and test sets for $P_{max}=6$.

If the FNN heuristics is included in the AE loss function formulation, a regularization term ϱ(z) is added to c(V,U) in accordance with Equation (3). As an outcome, Figure 19 reports the variance $\sigma (z_{p})$, with $p=1,\ldots ,P_{max}=6$, of each latent variable for the training set of configuration A, at varying value of the regularization parameter γ in the added term ϱ(z). Except the case $\gamma =10^{-3}$, for which the regularization term appears to be too small, $c_{FNN}(V,U,z)$ allows to automatically turn off some of the latent variables. Increasing values of γ are not associated to a clear trend in the number of deactivated latent variables. Indeed, the way in which the plain AE loss function c(V,U) and the regularization term γϱ(z) affect the solution is made practically unpredictable by the strong nonlinear behavior of $c_{FNN}(V,U,z)$.

**Figure 20:**
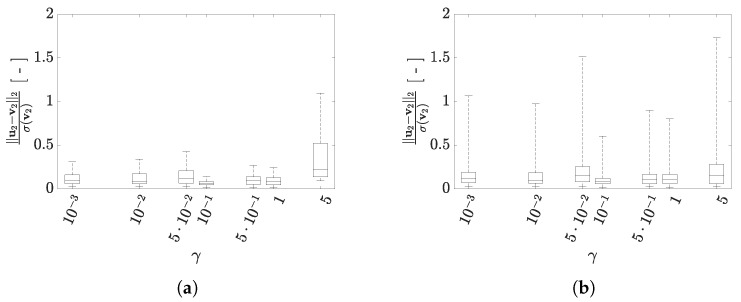

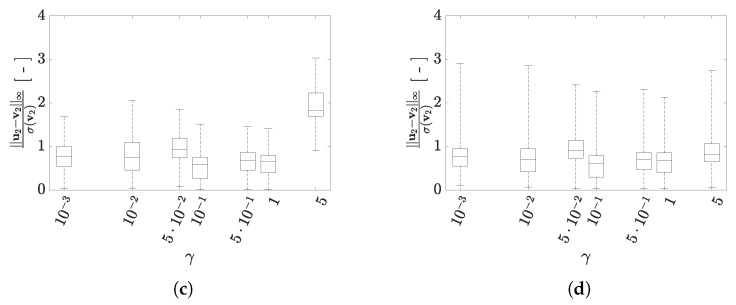
Two-storey shear building, configuration A. Effect of γ on the statistics of the reconstruction errors relevant to the floor displacement $v_{2}$, represented through box plots: (**top row**) standardized $L^{2}$ norm, and (**bottom row**) standardized $L^{\infty }$ norm; data taken from (**left column**) training set, and (**right column**) test set.

Even the reconstruction capacity of the AE does not show a clear trend at varying γ, as highlighted in Figure 20 in terms of the results obtained for the training and test sets relevant to configuration A. Though a non-monotonic variation of the AE performance is obtained, the FNN heuristics can be exploited to set the value of γ for which the mean value of the reconstruction error and the variation around it, are minimized. For configuration A, the minimum is obtained for $\gamma \approx 10^{-1}$, irrespective of the handled standardized norm and the dataset. For cases featuring such values of γ, the number of active latent variables is P=3, the same found as optimal using the standardized $L^{\infty }$ error norm. Even though they are not shown here for the sake of brevity, results relevant to configuration B are characterized by a slightly more regular effect of γ on the number of active variables in z, with a sub-optimal solution attained with the same range of values for the regularization term, endowed with P=4. In this case, the FNN heuristics has been able to attain a sub-optimal solution, but not the optimal one, featuring instead P=6.

The regularization of the loss function based on the FNN heuristics thus allows to basically achieve the same optimal AE settings already found. The strength of the regularization term seems to have a non negligible impact on the results. For this reason, the use of $c_{FNN}(V,U,z)$ leads to marginal advantages for the proposed cases study, given that the tuning of *P* is substituted by the tuning of γ. On the other hand, this approach can be considered useful if there is no a clear understanding on the number of generative factors.

#### 4.1.4. Load Identification

**Figure 21:**
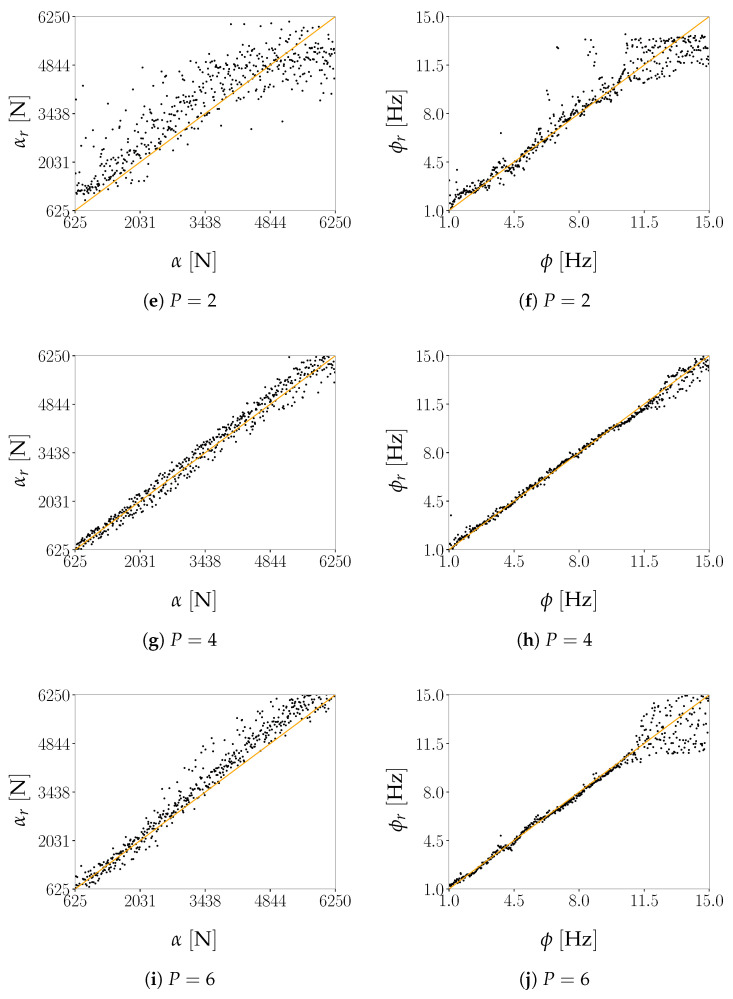
Two-storey shear building, configuration A, test set. Parity plots showing the regression outcomes for the load amplitude α (**left column**) and the load frequency ϕ (**right column**), at varying dimension *P* of the latent representation, against the ground-truth data.

As already pointed out, the latent representation z can be exploited to reduce the computational cost of solving inverse problems. For the present case, the identification of the loading parameters η on the basis of the relevant observations in V, has been approached by first exploiting the encoder function enc, to obtain z, and then by applying the regression model *r*. Main results are collected in Figure 21, where the effects of *P* on the identification of α and ϕ are reported for configuration A. In the graphs, the predicted values are reported along the vertical axis and are displayed against the corresponding ground-truth data reported along the horizontal axis: the perfectly identified values in each chart are therefore those aligned with the line $\alpha_{r}=\alpha$ or $\phi_{r}=\phi$. The accuracy of the regression model *r* is further assessed through Table 4, reporting the root mean square error (RMSE) between the predicted and the actual values, and the Pearson correlation coefficient $R^{2}$: when $R^{2}=1$, an exact linear correlation between predicted and ground-truth data is found, indicating the perfect alignment with the line $\alpha_{r}=\alpha$ or $\phi_{r}=\phi$.

The reconstruction accuracy of the AE has a direct impact on the regression accuracy, which is accordingly higher for P=4 and lower for P=2. A non monotonic dependence of the AE accuracy on the latent space size *P* is shown by the values of the RMSE (the lower, the better) and $R^{2}$ (the higher, the better) when both α and ϕ are targeted. Both indicators improve moving from P=2 to P=4, deteriorate for P=5, get better for P=6 but still without reaching the performance for P=4. These results are in agreement with the box plots in Figure 16 and, partially, with the indications of FNN heuristics, pointing out P=4 as the optimal dimension for the latent representation. Regarding the load frequency ϕ, the regressor *r* faces some difficulties in the frequency range within which also the reconstruction capacity of the AE has been shown to be detrimentally affected. Indeed, by comparing Figure 10e and Figure 21f, the scattering in the prediction for $\phi >f_{2}$ corresponds to the high error measured via the standardized $L^{\infty }$ norm.

### 4.2. Pirelli Tower

**Figure 22:**
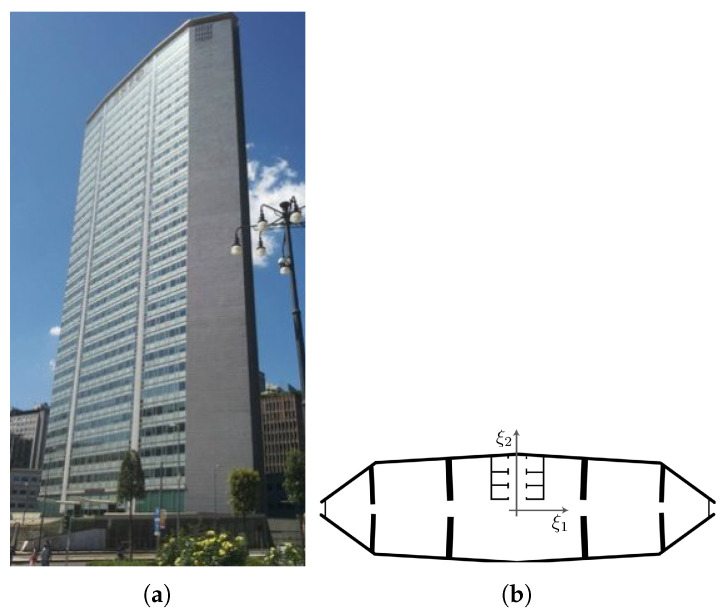
The Pirelli Tower in Milan. (**a**) Picture taken from Piazza Duca D’Aosta, and (**b**) schematic plan of a standard floor.

A real-life case is now considered, to get more insights regarding the capability of the proposed approach: the Pirelli Tower in Milan (see Figure 22a). This tower is a 39 storey building, 35 of which out of ground, having a total height of 130 m. A schematic representation of the standard floor, whose dimensions are approximately 70×20 m, is reported in Figure 22b. Due to its constant plan geometry, a shear building model was proven to describe well the dynamic response of the tower under horizontal actions: the floors behave like rigid diaphragms, connected through compliant columns modelling the lateral load resisting system. The soil-structure interaction at its basement has been modeled via a lumped-parameter approach: additional details were thoroughly reported in [40].

To slightly simplify the analysis, torsional effects due to the small eccentricity of the center of stiffness with respect to the center of mass along the $\xi_{2}$ axis, as induced by the asymmetric central core, have been disregarded. The lateral loads given by Equation (4) have been applied along the $\xi_{1}$ direction; the amplitude α has been sampled from the uniform pdf $U_{\alpha }(2\times 10^{3},20\times 10^{3})\;N$; the load frequency ϕ has been instead sampled from three uniform pdfs $U_{\phi }^{1}(1,6)$ Hz, $U_{\phi }^{2}(1,9)$ Hz, and $U_{\phi }^{3}(1,15)$ Hz, respectively, defined as load cases 1, 2 and 3. The load case 3 features the same frequency range used for the two-storey building; load cases 1 and 2 have been instead designed aiming to ease the load identification task, as can be ascertained by comparing the relevant frequency ranges with the fundamental vibration ones of the building, gathered in Table 5.

A pseudo-experimental monitoring frame has been adopted, assuming that the lateral displacements at the 20th and at the 39th floors are recorded for T=5 s, with a sampling frequency of 50 Hz to avoid aliasing in relation to the first 13 vibration modes of the structure. An optimal placement of the sensors to monitor the health of this building was discussed in [42], by exploiting a Bayesian experimental design that requires a detailed description of the parameter uncertainties. An optimization of sensor placement based on non-probabilistic interval analysis may be instead preferable when such a description of the uncertainties can not be fully provided [47]. To train the AE 32,000 MTS have been generated, still keeping 75% of the samples for training and 25% of them for validation. The number of instances have been doubled with respect to the two-storey case, due to the greater complexity of the present model. Regarding computational costs and resources, an RTX 2080 Ti (GPU) has been exploited for both the training and the testing stages. For the case at hand, the training of the AE has lasted 1 h and 50 min, while the training of the regressor *r* has lasted about 10 min. The testing has resulted instead almost inexpensive, since each instance is processed in about 0.05 s.

**Figure 23:**
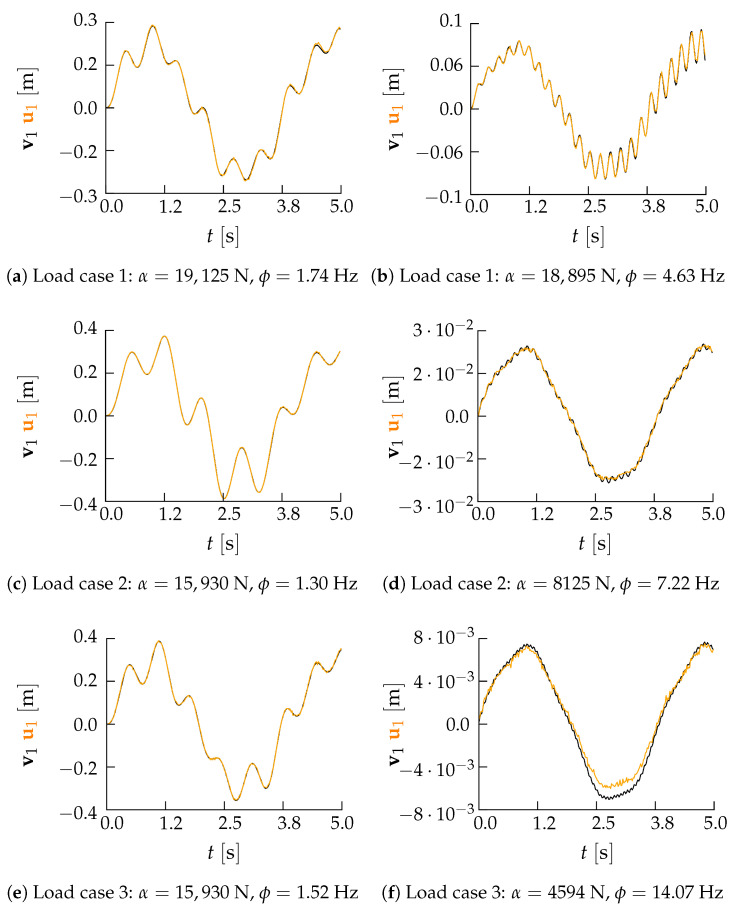
Pirelli Tower. Comparison between input (black lines) and reconstructed (orange lines) time histories of the 20-th floor displacement for six cases belonging to the test set.

To qualitatively assess the reconstruction capacity of the AE when test instances are considered, results are reported in Figure 23 in terms of the histories of the 20th floor displacement for a couple of solutions related to each load case. For each case, the reconstructed signal has resulted close to the input one for low excitation frequencies, but the accuracy seems to get reduced by larger values of ϕ. This outcome is again related to what discussed in Section 4.1.2 regarding the mode factors $\Gamma_{s}$, computed similarly to Equation (11) for s=1,…,39, which are larger for the low-order vibration modes.

**Figure 24:**
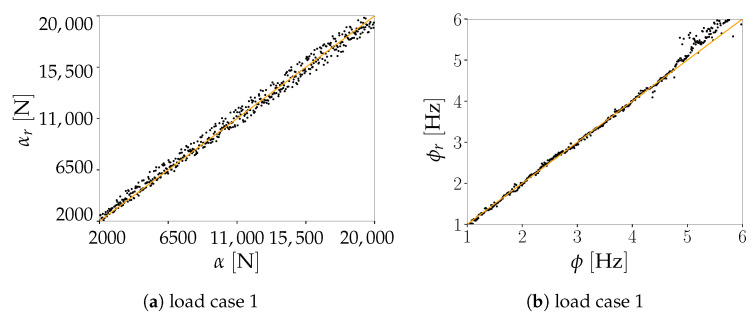

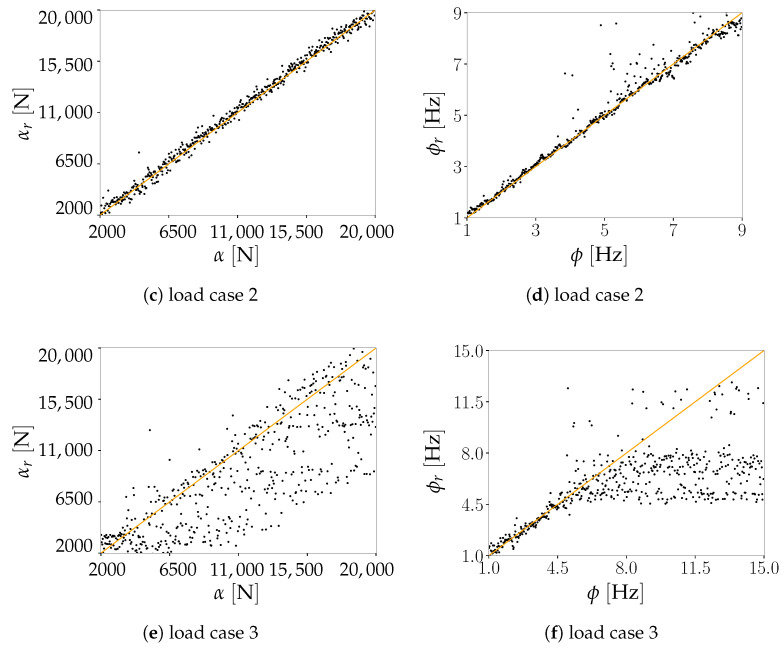
Pirelli Tower. Parity plots showing the regression outcomes for the load amplitude α (**left column**) and the load frequency ϕ (**right column**) for the three considered load cases.

Regarding load identification, outcomes are reported in Figure 24 and in Table 6. As shown for the two-storey shear building, the reconstruction accuracy of the AE has an impact on the performance of the regressor *r*. For ϕ>5 Hz, the regression outcomes of the load cases 1 and 2 get slightly deteriorated, with an increased scattering of the values of $\phi_{r}$ for load case 2 as assessed by the relevant values of the RMSE (passing from 0.144 Hz to 0.417 Hz) and of the Pearson correlation coefficient $R^{2}$ (moving down from 0.998 to 0.984). For those two load cases, the load amplitude has been always well predicted, with errors bounded independently of α. Hence, the AE reconstruction capacity is only marginally affected by the load amplitude.

A different type of outcomes has been obtained for the load case 3. Focusing on $\phi_{r}$, the regression task fails if ϕ>9 Hz. The Pearson correlation coefficient $R^{2}$ falls from almost unitary values for the load case 1 and 2 to 0.808 for the load case 3 when the identification of α is addressed, and from 0.984 for load case 2 to 0.679 when ϕ is targeted. A way to explain this behavior is again linked to the small values of $\Gamma_{s}$ associated to higher vibration modes; accordingly, the critical part of the procedure is still assumed to be due to signal reconstruction. Moreover, in comparison to the load cases 1 and 2, the regression performance gets spoiled in the frequency range 5.5 Hz<ϕ<9 Hz. The predicted values exhibit a sort of cut-off around 8 Hz, above which it becomes impossible to obtain reasonable predictions. The complexity of the AE loss c(V,U) hence forces the training algorithm to converge to a local minimum that worsens the regression performance, not only within the previously mentioned range but also for all the values larger than 6 Hz. A reduced performance has been also shown regarding the load amplitude α, even if a cut-off has not been reported. Overall, the regressor tends to systematically underestimate α, except for the instances featuring ϕ<5 Hz which are instead caught correctly.

**Figure 25:**
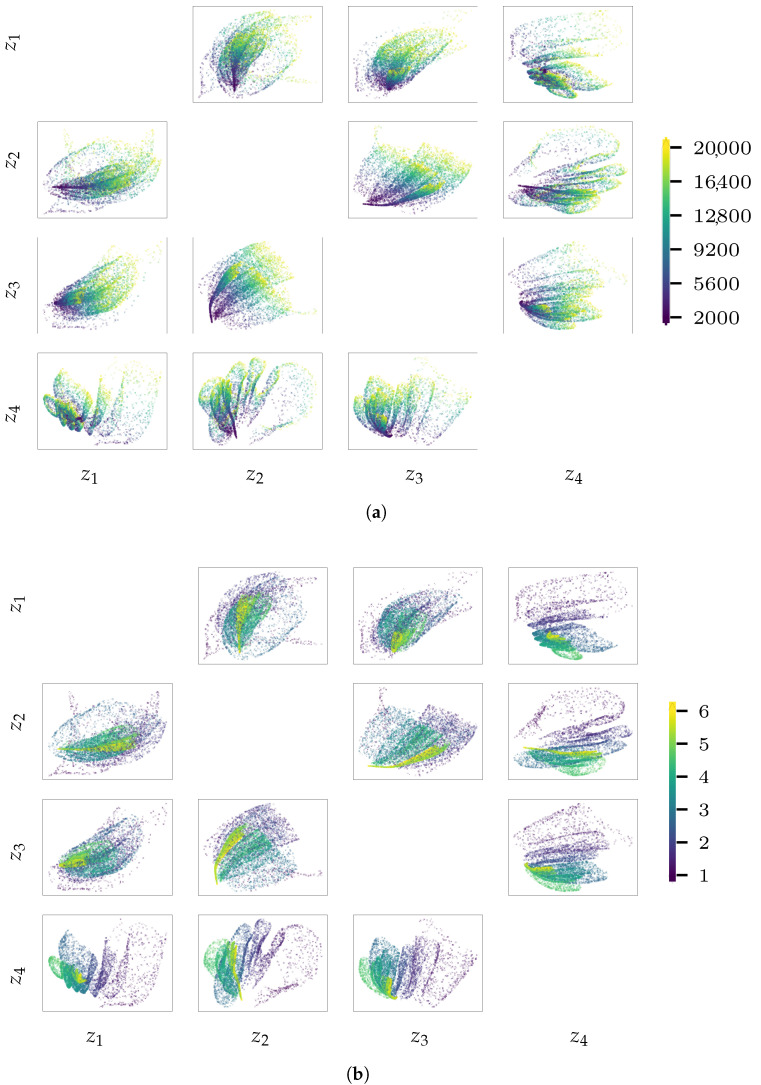
Pirelli Tower, load case 1. Scattered latent representation z determined by enc for the validation set, with coding set by (**a**) the load amplitude α or by (**b**) the load frequency ϕ.

**Figure 26:**
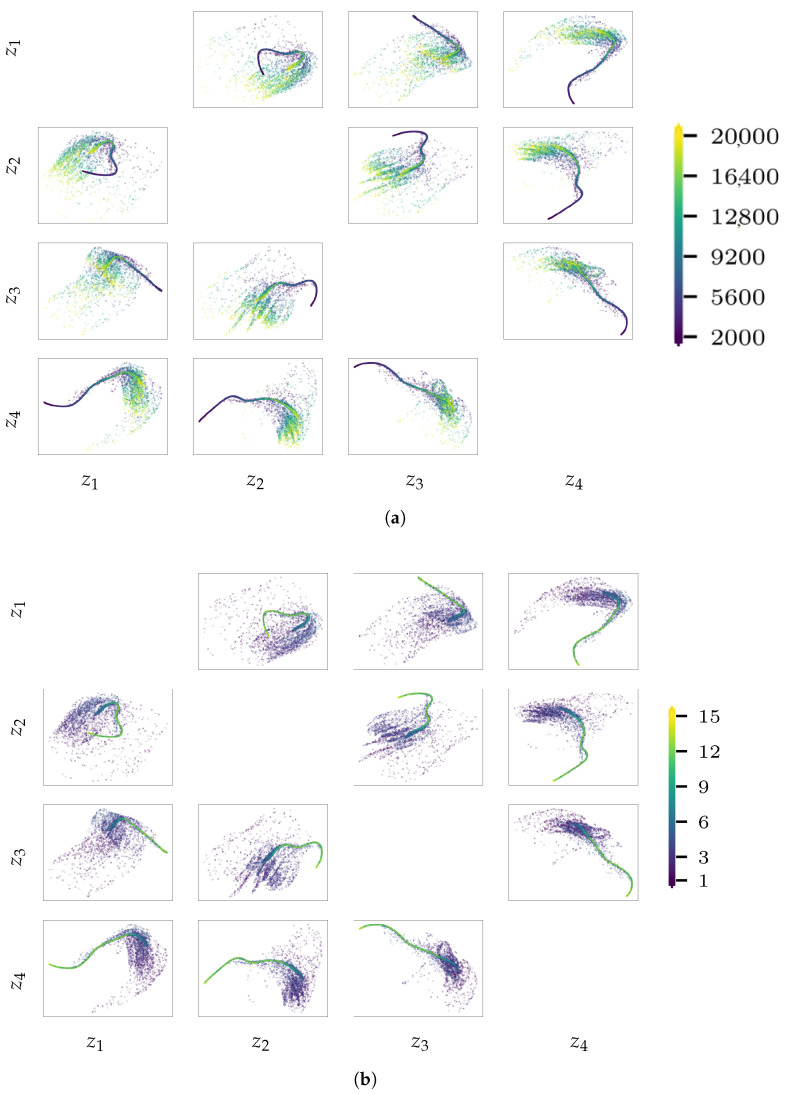
Pirelli Tower, load case 3. Scattered latent representation z determined by enc for the validation set, with coding set by (**a**) the load amplitude α or by (**b**) the load frequency ϕ.

To address the above discussed issues, we report in Figure 25 and Figure 26 the latent variables z for the validation sets of load cases 1 and 3. The focus is only on the out-of-diagonal dispersion plots in this matrix-like representation of the results, wherein each chart shows the values taken by pairs of latent variables. A color code is used in all the plots, to visualize how the generative factors α and ϕ affect the encoding distribution in the latent space. These results confirm that the load identification issues are due to the encoding procedure: looking at Figure 26b, it is clearly shown that when ϕ is larger than the cut-off-value discussed in relation to Figure 24f, the encoded values are not spread anymore in the sub-space spanned by the said pairs of latent variables. For a frequency in the range 5 Hz<ϕ<8 Hz, the encoded states start to show a tendency towards collapsing into lower-dimensional loci, though a small scattering is still present and the regressor can predict load frequencies in this range. We can thus state that the greater the scattering of the latent variables, the more effective the encoded representation in terms of signal reconstruction and load identification. This is confirmed by looking at the results related to load case 1: the best reconstructed instances are obtained for the most scattered encodings, characterized by small values of ϕ. When the encodings become less scattered, they also become less informative for the signal reconstruction. By looking at Figure 25, it can be also noticed that the instances featuring both larger values of α (reported in yellow in the plots) and smaller values of ϕ (reported in violet in the plots) are those farthest from the collapsed loci.

## 5. Conclusions

A time series AE has been designed and adopted to solve a regression task linked to load identification, which plays a crucial role in the assessment of the health of a structure if operational variability is allowed for, or in case of an output-only architecture of the monitoring system. The role of the latent representation provided by the AE has been extensively discussed, allowing for the sources of output variability of the tackled problem and the inverse-forward nature beyond the autoencoding paradigm.

For a two-storey shear building model, the reconstruction capacity of the AE has been quantitatively assessed by using two error norms, and by investigating the effect on it of the structural vibration frequencies and modes. Physically-sound links with the dynamics of the system have been shown, pinpointing how the excited structural vibrations can play a key role in setting the AE outcomes. The false nearest neighbor heuristics has been also allowed for to automatically set the dimension of the latent representation. The results obtained for the two-storey shear building have been then taken into account to approach load identification for a digital twin of a high-rise building, the Pirelli Tower in Milan. Promising outcomes have been reported, to foresee future applications of the proposed methodology to real-life situations.

The next steps will be to further empower the setting procedure for the AE hyperparameters, by combining the here adopted trial and error procedure with Bayesian methods [48]. Optimization methods for the deployment of the sensors of the monitoring system will be also proposed, in order to maximize the information content of the latent representation and, at the same time, minimize the number of sensors to deploy and avoid issues linked to big data (see, e.g., [52]).

## Figures and Tables

**Table 1:** Two-storey shear building model: data relevant to configurations A and B, see Figure 7.

		Configuration A	Configuration B
$m_{1},m_{2}$	(ton)	625, 625	625, 1250
$k_{1},k_{2}$	($\frac{\mathrm{kN}}{m}$)	$10^{6},10^{6}$	$10^{6},\;3\times 10^{6}$
$f_{1},f_{2}$	(Hz)	3.93, 10.3	3.41, 14.5

**Table 2:** Adopted error measures, where $\sigma (v_{n})$ is the standard deviation of the TS $v_{n}$.

standardized $L^{2}$ norm	$\frac{\left|\right|u_{n}-v_{n}{\left|\right|}_{2}}{\sigma (v_{n})}$
standardized $L^{\infty }$ norm	$\frac{\left|\right|u_{n}-v_{n}{\left|\right|}_{\infty }}{\sigma (v_{n})}$

**Table 3:** Two-storey shear building model: AE hyperparameters.

Encoder		Decoder	
$\left[N_{e}^{inc_{1}},N_{e}^{inc_{2}},N_{e}^{inc_{3}}\right]$	[3N,3N,6N]	$N_{d}$	48N
$\left[H_{e}^{inc_{1}},H_{e}^{inc_{2}},H_{e}^{inc_{3}}\right]$	[13,8,5]		
$N_{e}^{2}$	6N		
$\left[H_{e}^{1},H_{e}^{2},H_{e}^{3}\right]$	[8,5,3]		

**Table 4:** Two storey shear building, configuration A, test set. RMSE and correlation coefficient $R^{2}$ of the regression outcomes for the load amplitude α and the load frequency ϕ, at varying dimension *P*.

P	α		ϕ	
	RMSE [N]	$R^{2}$ [-]	RMSE [Hz]	$R^{2}$ [-]
2	1263	0.654	1.74	0.912
3	1243	0.717	1.11	0.963
4	552	0.941	0.69	0.987
5	1004	0.803	1.16	0.960
6	769	0.897	1.28	0.966

**Table 5:** Fundamental vibration frequencies of the Pirelli tower.

Vibration Mode	Frequency f (Hz)
1	0.25
2	1.08
3	2.60
4	4.71
5	7.06
6	8.79
7	9.56
8	9.91
9	11.38
10	13.36
11	14.64
12	18.30
13	22.14

**Table 6:** Pirelli tower. RMSE and correlation coefficient $R^{2}$ of the regression outcomes for the load amplitude α and the load frequency ϕ for the three considered load cases.

Load	α		ϕ	
Case	RMSE [N]	$R^{2}$ [-]	RMSE [Hz]	$R^{2}$ [-]
1	469	0.996	0.144	0.998
2	439	0.997	0.417	0.984
3	3852	0.808	3.758	0.679

## Data Availability

The numerical cases of study have been exhaustively described. The reader may generate the same data used in this paper.
